# Novel role for non-invasive neuromodulation techniques in central respiratory dysfunction

**DOI:** 10.3389/fnins.2023.1226660

**Published:** 2023-08-23

**Authors:** Lan Lv, Xiaoping Cheng, Jiaying Yang, Xinyuan Chen, Jun Ni

**Affiliations:** Department of Rehabilitation Medicine, The First Affiliated Hospital of Fujian Medical University, Fuzhou, China; ^2^Department of Rehabilitation Medicine, National Regional Medical Center, Binhai Campus of the First Affiliated Hospital, Fujian Medical University, Fuzhou, China

**Keywords:** non-invasive neuromodulation techniques, respiratory dysfunction, TMS, tDCS, central

## Abstract

Respiration is a crucial steady-state function of human life. Central nervous system injury can damage the central respiratory pattern generator (CRPG) or interrupt its outflow, leading to central respiratory paralysis and dysfunction, which can endanger the patient's life. At present, there is no effective means to reverse this process. Commonly used non-invasive neuromodulation techniques include repetitive transcranial magnetic stimulation (rTMS), transcranial direct current stimulation (tDCS) and so forth, which have been widely applied in nervous system diseases and their various secondary symptoms, but rarely in respiratory function. Clinical and animal studies have confirmed that TMS is also suitable for investigating the excitability and plasticity of ascending corticospinal respiratory pathways. In addition, although rTMS and tDCS differ in their respective mechanisms, both can regulate respiratory networks in healthy individuals and in diseased states. In this review, we provide an overview of the physiology of respiration, the use of TMS to assess the excitability of corticophrenic pathways in healthy individuals and in central respiratory disorders, followed by an overview of the animal and clinical studies of rTMS, tDCS and so forth in regulating respiratory circuits and the possible mechanisms behind them. It was found that the supplementary motor area (SMA) and the phrenic motor neuron (PMN) may be key regulatory areas. Finally, the challenges and future research directions of neuroregulation in respiratory function are proposed. Through understanding how neuromodulation affects the respiratory neural circuit non-invasively, we can further explore the therapeutic potential of this neuromodulation strategy, so as to promote the recovery of respiratory function after central nervous system diseases or injury.

## Introduction

Respiration is a crucial steady-state function of human life. Central nervous system injury can damage the central respiratory pattern generator (CRPG) or interrupt its outflow, such as in stroke, high cervical spinal cord injury and so forth, thus leading to central respiratory paralysis and dysfunction, which can endanger the patient’s life. At present, there is no effective means to reverse this process, and the commonly used methods are alternative, such as mechanical ventilation support, oxygen therapy and so forth. Neuromodulation techniques are defined as “the alteration of nerve activity through targeted delivery of a stimulus, such as electrical stimulation or chemical agents, to specific neurological sites in the body” (Knotkova et al., 2021). Commonly used non-invasive neuromodulation techniques include repetitive transcranial magnetic stimulation (rTMS) and transcranial direct current stimulation (tDCS), which have been widely applied in nervous system diseases and their various secondary symptoms, but rarely in respiratory function.

Clinical and animal studies have confirmed that TMS is also suitable for investigating the excitability and plasticity of ascending corticospinal respiratory pathways (Urban et al., 2002; Miscio et al., 2006; Harraf et al., 2008; Vinit et al., 2014; Lee et al., 2021). Generally, TMS relies on the generation of a strong magnetic field through an electromagnetic coil, which creates an electric current that travels approximately 3 cm through the cerebral cortex and depolarizes the cortical neurons (Lefaucheur et al., 2014; Kubis, 2016). Repetitive TMS (rTMS) involves a series of continuous or periodic pulses that are thought to induce long-term changes in cortical excitability. The mechanism of cortical excitability changes is not fully understood, but some researchers believe that it is similar to long-term potentiation (LTP) and long-term depression (LTD) (Bates and Rodger, 2015; Kubis, 2016). However, there are arguments that rTMS mediated effects are usually the result of a mixture of synaptic events (Kemps et al., 2022). The mechanism underlying tDCS is also not fully understood. Unlike the direct induction of neuronal activity by TMS, tDCS is thought to have a main mechanism involving the subliminal regulation of neuronal membrane potential, which biases cortical excitability (Nitsche and Paulus, 2000). The direct effect of tDCS on corticospinal excitability is mainly determined by changes in subliminal resting membrane potential (Nitsche et al., 2005). In addition to acute effects on brain function, specific protocols are suitable for inducing long-lasting alterations in cortical excitability and activity, which share features with long-term potentiation and depression (Stagg et al., 2018). However, a cadaver study doubts the mechanism and effectiveness of tDCS, and Underwood (2016) suggests that 1–2 mA currents are unlikely to have dramatic effects on neurons. Although the mechanisms of rTMS and tDCS are different, both can alter respiratory excitability by stimulating relevant regions (Raux et al., 2010; Azabou et al., 2013; Laviolette et al., 2013; Niérat et al., 2014; Nierat et al., 2015; Carvalho et al., 2021). Through understanding how neuromodulation affects the respiratory neural circuit non-invasively, we can further explore the therapeutic potential of this neuromodulation strategy, to promote the recovery of respiratory function after central nervous system diseases or injury. In the following section, we provide an overview of TMS in evaluating the excitability of the cortico-diaphragmatic pathway (Supplementary Table 1), animal and clinical studies on rTMS and tDCS regulating respiratory function (Supplementary Tables 2, 3), as well as the difficulties encountered and future directions for development.

## Physiology of respiration

The central control of respiration is dual, automatic at the brainstem level and voluntary at the cortical level. The preBötzinger complex (preBötC) of the medulla oblongata underlies automatic inspiratory rhythm generation, while the retrotrapezoidnucleus/parafacial respiratory group (RTN/pFRG) generates active expiration (Feldman et al., 2013). Autonomous respiratory command originates in the cerebral cortex, and is transmitted to respiratory motor neurons in the spinal cord through the medullary reticulospinal tract and corticospinal tract, among which phrenic motor neurons (PMNs) are the main respiratory motor neurons located in the cervical spine (C3–C5) (Verin et al., 2011). The contraction of the diaphragm depends on the PMN discharge. Functional magnetic resonance imaging (fMRI) studies have shown that the sensory-motor cortex, cerebellum, supplementary motor area (SMA) and premotor area are mostly activated in neuroimaging for respiratory motor control, while additional respiratory motor activities are detected in the basal ganglia, thalamus and prefrontal cortex by high-sensitivity neuroimaging (Evans, 2010).

## Preclinical studies on examination and neuromodulation of respiration

Respiratory dysfunction associated with neural control is life-threatening. To date, there is no effective treatment method for improving damaged function. Therefore, it is important to establish a preclinical model for further respiratory research to develop a non-invasive therapeutic tool suitable for animal phrenic neural circuits. Vinit et al. (2014) firstly established an animal model of TMS-induced diaphragmatic motor evoked potentials (DiMEPs) in Sprague–Dawley (SD) rats for trans-synaptic neuroanatomical tracing with pseudorabies virus (applied to the diaphragm), which revealed that supraspinal stimulation could (directly or indirectly) transmit anatomical substrates of descending action potentials to spinal motor neurons. The authors further applied it to the study on respiratory plasticity in rats with respiratory dysfunction following C2 hemisection, and observed profound reorganization in the TMS-induced diaphragm. DiMEPs decreased on the non-injured side, but not the injured side, indicating increased excitability of PMNs on the ipsilateral side. In addition, there was a correlation between the DiMEP amplitude and spontaneous contralateral diaphragmatic activity. The higher the degree of diaphragmatic activity, the higher the DiMEPs on the injured side, and the lower the DiMEPs on the non-injured side. This suggests, for the first time, the occurrence of a functional neuroplasticity process involving changes in motoneuron excitability balance between the injured and the non-injured sides in a short time after injury (Vinit et al., 2016). On this basis, Michel-Flutot et al. (2021) conducted two interventional studies. One study compared different rTMS protocols and found that the 10-Hz rTMS protocol induced a sustained and stable increase in the excitability of PMNs compared with 3 Hz and 30 Hz. Another study investigated the effect of chronic high-frequency (10 Hz) rTMS on the cortical regions of rats with C2 hemisection. One month after treatment with 7-d, 1-month or 2-month rTMS, an increase in activity and excitability (DiMEPs) was observed on the non-injured side in diaphragmatic electromyography (EMG). Interestingly, although rTMS treatment did not have an actual functional impact on damaged diaphragmatic activity during respiratory stimulation, a 2-month rTMS treatment strengthened the existing crossed phrenic pathway, increasing the activity of the damaged diaphragm during respiratory stimulation. These findings demonstrate that chronic high-frequency rTMS can improve respiratory dysfunction after cervical spinal cord injury, and that this therapeutic tool can be used and/or combined with other interventional measures to further improve beneficial clinical outcomes (Michel-Flutot et al., 2022). In addition to inducing DiMEPs through TMS, some scholars have conducted animal studies on trans-spinal magnetic stimulation. When Lee et al. (2021) placed the rat head 30 mm right or left to the coil center, and a single magnetic stimulation could induce significant DiMEPs in non-injured animals. In the acute stage of left cervical spinal cord injury, cervical magnetic stimulation reduced the DiMEP threshold and enhanced its amplitude. In addition, during the subchronic and chronic stages, the bilateral DiMEPs of the contused animals increased when the coil was placed in the left cervical spinal cord, suggesting that cervical magnetic stimulation can be used to detect the excitability of diaphragmatic motor output after injury, and the more lateral the magnetic stimulation direction, the better the effect of triggering DiMEPs. In another study, Lee et al. (2022) explored the rostral-caudal effect of spinal cord magnetic stimulation on DiMEPs following cervical spinal cord injury, and the effects of coils at the rostral, middle and caudal levels of rats. The results showed that cervical magnetic stimulation could induce intensity-dependent MEP in the bilateral diaphragm of both normal rats and rats with left cervical spinal cord contusion, but the amplitude of the left diaphragm was higher and its occurrence was earlier than that of the right. Moreover, the intensity-response curve of magnetic stimulation shifted upwards in the rostral-caudal direction, indicating that caudal cervical magnetic stimulation generated higher DiMEPs than rostral cervical magnetic stimulation. After cervical magnetic stimulation, the DiMEPs of contused rats were similar to that of normal rats, but the diaphragmatic inspiratory activity of contused rats was weaker. Additionally, in rats with contusion, the amplitude of DiMEPs in the chronic stage was higher than that in the early stage.

However, the respiratory physiology of humans and animals is not entirely consistent. Rats have specific respiratory medullary spinal axons with C3-C6 spinal cord segments crossing the midline, known as the crossed phrenic pathway (Goshgarian, 2003), which has not been observed in humans. Further research is required to determine whether the results of animal models are applicable to humans.

## Evaluation of cortico-diaphragmatic spinal pathway and respiration-related cortical excitability using TMS

TMS has been used to characterize the motor cortex of the diaphragm and evaluate the cortico-diaphragmatic pathways in both hemispheres. Murphy et al. (1990) first used non-focal TMS to determine the optimal location for eliciting DiMEPs, centered on a circular coil with an average of 2 cm posterior to the vertex in the median sagittal line, where bilateral diaphragmatic responses could be induced. Subsequently, it was recorded that under TMS, a focal 8-shaped coil mainly caused contraction of the contralateral diaphragm with its center approximately 3 cm right to the midline and 2 ± 3 cm in front of the auricular plane, however, small EMG responses were also observed on the ipsilateral side. The bilateral corticospinal cord and the diaphragm have crossed and uncrossed connections, mainly crossed tracts (Maskill et al., 1991; Khedr and Trakhan, 2001). All the above stimuli corresponded to the primary motor cortex of the diaphragm. Sharshar et al. (2003) found that TMS of the supplementary motor area (SMA)in front of the primary motor cortex of the diaphragmcan also elicit DiMEPs. During spontaneous inspiration, the two cortical regions that can lead to diaphragmatic responses showed significant differences in inhibitory/excitatory balance and output facilitation in the cortex, suggesting another SMA-diaphragm conduction pathway. The authors concluded that SMA might play a major excitatory role on PMNs.

Clinically, TMS has been used to record respiratory muscle involvement in patients with nervous system diseases. It has been shown that the excitability threshold of the diaphragmatic cortex decreases and the conduction time of the diaphragm and intercostal muscle involvement pathways are prolonged in patients with stroke (Khedr et al., 2000; Urban et al., 2002). In addition to the inspiratory muscles, a TMS study on expiratory muscle weakness in acute ischemic stroke was conducted using TMS at the vertex (a representative area of the diaphragmatic cortex) and bilateral hemispheric expiratory muscular cortex, as well as magnetic stimulation over the T 10–11 spinal roots (Tw T10) and phrenic nerves bilaterally (BAMPS), with surface electrodes recording MEPs of the rectus abdominis (RA) and external oblique (EO). The results revealed that the latency and amplitude of MEPs induced by TMS in the abdominal muscles in the uninjured hemisphere of patients with stroke were comparable to those in the control group, but no MEPs were recorded in the abdominal muscles after TMS of the injured hemisphere. TMS at the cortical area of the expiratory muscles in the injured hemisphere resulted in lower intragastric pressure compared to the uninjured side, suggesting that ischemic cortical injury is correlated with expiratory muscle weakness and may cause cough in stroke patients with acute respiratory failure (Harraf et al., 2008). TMS has also been used to explore cortico-diaphragmatic pathways in patients with amyotrophic lateral sclerosis (ALS), with no changes in vital capacity or blood gas levels in all 14 patients. Seven patients had a decrease in maximal transdiaphragmatic pressure (Pdimx), and eight patients showed a decrease in MEPs. Four patientshad delayed spinal motor-evoked potentials (Sp-MEPs). Cortical motor-evoked potentials (Cx-MEPs) were not elicited in one patient. The correlations between Cx-MEPs and central motor conduction time (CMCT) with any respiratory measurement were not significant, indicating that cortico-diaphragmatic research is a sensitive method to reveal subclinical diaphragmatic injury, although it is not correlated with respiratory measurements (Miscio et al., 2006). Similar studies have also been carried out in multiple sclerosis (MS), and the results showed prolonged Cx-MEP latency and CMCT in the diaphragm (Dia) of 31 and 23% respectively, as well as in the abductor digiti minimi (Abd) of 76 and 79% patients. Phrenic nerve-compound motor action potentials (PN-CMAPs) were normal. This suggests that the cortico-diaphragmatic pathways are damaged only in a few MS patients (Miscio et al., 2003). Furthermore, DiMEPs have also been used to predict whether patients with respiratory failure can be weaned from the ventilator. Once study followed up the DiMEPs of ventilator-dependent patients due to central respiratory failure, and found that the MEPs were all restored in patients who were able to wean from the ventilator, while those who could not wean did not. This suggests that TMS can play a role in predicting recovery of respiratory function in central respiratory paralysis (Duguet et al., 2006).

## Non-invasive neuromodulation techniques as interventions in respiration

The cortical motor center of the diaphragm is considered to be located at the vertex (circular coil) or approximately 3 cm right to the midline and 2 ± 3 cm in front of the auricular plane (8-shaped coil), and the SMA is considered to have another conduction pathway between the diaphragm. Interestingly, several stimulus locations for TMS-based neuromodulation of respiration in healthy individuals are all located in the SMA. A TMS study revealed that 5-Hz rTMS acting on the SMA simultaneously increased MEP amplitude in the diaphragm and the first dorsal interosseous (FDI) muscle, suggesting that changing SMA excitability can cause excitability changes in the diaphragmatic motor cortex.However, 1-Hz rTMS could not reduce the MEP amplitude of the diaphragm or FDI muscle (Raux et al., 2010). However, in another clinical study, magnetic stimulation of the SMA bi-directionally regulated the corticospinal pathway of the diaphragm, and continuous theta burst stimulation (cTBS, inhibitory) of the SMA during quiet and natural respiration suppressed the excitability of the corticospinal pathway to the diaphragm. The excitatory repetitive magnetic stimulation (5 Hz, excitatory) paradigm applied to the SMA enhanced the excitability of the corticospinal pathway to the diaphragm (Laviolette et al., 2013). In addition to changing the excitability of the diaphragmatic motor cortex, magnetic stimulation of the SMA can also alter respiratory pattern.One study confirmed that controlling SMA excitability through rTMS could alter the respiratory response mode to experimental inspiratory load and may improve respiratory discomfort. The 5-Hz pre-treatment scheme could reduce the excessive ventilation caused by the inspiratory threshold load. Inhibitory pre-treatment did not affect ventilation, but prolonged expiratory time. After sham stimulation, there were no significant changes in the respiratory pattern to inspiratory load (Nierat et al., 2015). Currently, TMS in regulating patients with respiratory dysfunction has rarely been reported. A newly reported clinical study on the application of TMS in stroke patients with respiratory dysfunction found that rTMS acting on the diaphragmatic cortical center five times a week for 8 weeks could improve pulmonary function after acute ischemic stroke. However, this study did not measure DiMEPs;therefore it cannot confirm the direct effect of TMS on the diaphragm (Cao et al., 2022).

tDCS has also been used to regulate the respiratory centers. In a study about the effect of tDCS on the diaphragmatic corticospinal pathway in healthy individuals, anode, cathode and sham tDCS were randomly applied to the left diaphragmatic motor cortex of 12 healthy right-handed males. The excitability of the corticospinal pathway was evaluated using TMS-induced MEPs. The results showed that the excitability of the diaphragmatic corticospinal pathway decreased regardless of polarity (Azabou et al., 2013). In a recent clinical case report(Carvalho et al., 2021), the authors selected tDCS on SMA combined with peripheral electrical stimulation (PES) based on the results of high-frequency TMS research. Corticospinal excitability may also be affected by PES, depending on the parameters used. Sensory PES is often inhibitory, while motor PES is usually excitatory (Chipchase et al., 2011a,b). The authors reported two cases of SCI (P1 and P2) with long-term tracheotomy (>40 days) and hospitalization (>50 days). P1 received the combined application of sensory PES on the pectoral and abdominal muscles and anode tDCS on the SMA, while P2 received isolated excitatory PES on the abdominal muscles. Both patients were extubated 15 times after stimulation, and presented clinical effects such as cough effectiveness. This suggests that the SMA, under both TMS and tDCS, may be a key area for respiratory regulation, but this still needs to be confirmed in clinical trials with a large sample size.

The spinal motor center of the diaphragm is considered another regulatory target. Data from a randomized clinical trial showed that both anode and cathode transcutaneous spinal direct current stimulation (tsDCS) at the C3–C5 levels induced a progressive increase in DiMEP amplitude during stimulation, lasting at least 15 min after the end of stimulation. Interestingly, tsDCS induced a sustained increase in tidal volume at the cathode rather than at the anode. However, the long-term increase in tidal volume after cathode tsDCS is particularly noteworthy, as this finding paves the way for therapeutic research to evaluate tsDCS as a tool for increasing ventilation in patients with various neurorespiratory diseases (Niérat et al., 2014).

## Underlying mechanisms

### Plasticity of the respiratory network

In recent years, increasing evidence has shown that respiratory rhythm generation networks exhibit high plasticity. Although spontaneous functional recovery after cervical hemisection is limited, inducing additional plasticity (such as repeated exposure to intermittent hypoxia) can significantly enhance the respiratory motor outputin experimental models of cervical spine injury (cervical hemisection). The longer the duration after injury, the stronger the ability to induce functional recovery (Vinit et al., 2009; Dale-Nagle et al., 2010a; Lovett-Barr et al., 2012). Training can also alter respiratory plasticity. Diaphragm training leads to a decrease in the threshold intensity of stimulation, an increase in the number of responding sites mapped to the diaphragm under focal stimulation, and shortened latency of MEPs in response to non-focal stimulation (Demoule et al., 2008). The increased respiratory motor output induced by various factors is called phrenic motor facilitation (PMF). The most widely studied form of PMF is phrenic long-term facilitation (pLTF) after acute intermittent hypoxia (AIH). At least five different cellular mechanisms generate long-lasting phrenic motor facilitation (pMF) with similar intensity and time domains, including the Gq pathway. The Gq pathway is the “classic” mechanism of diaphragmatic LTF in anesthetized rats, initiated by the intermittent activation of 5-hydroxytryptaminergic neurons in the median raphe, which activates 5-hydroxytryptamine (Gq-coupled) type 2 metabotropic receptors located at or near PMNs. The downstream intracellular cascade of 5-hydroxytryptamine 2 receptor activation includes the new synthesis of brain-derived neurotrophic factor (BDNF) and the activation of its high-affinity receptor tyrosine kinase B (TrkB), followed by the activation of extracellular regulated protein kinases /mitogen-activated protein(ERK/MAP)kinase. The Gs pathway, which triggers similar pMF through a unique mechanism, requires the synthesis of new TrkB (rather than BDNF) and the activation of agammaglobulinaemia tyrosine kinase(Akt)(rather than ERK MAP kinase). In addition, other mechanisms include activation of the spinal vascular endothelial growth factor (VEGF) receptor, erythropoietin and persistent diaphragm inactivity (Dale-Nagle et al., 2010a,b, 2012; Mahamed et al., 2011). Inflammation induced by low-dose lipopolysaccharide may undermine mAIH-induced pLTF (Marciante and Mitchell, 2023).

### Transcranial direct current induces respiratory excitability

tDCS can induce sustained changes in excitability in the human motor cortex. Transcranial magnetic stimulation showed an approximately 150% increase in motor cortical excitability from baseline up to 90 min after stimulation ended (Nitsche and Paulus, 2001). Anode stimulation selectively increases cortical excitability, whereas cathode stimulation selectively reduces cortical excitability. A study found that the post-stimulation effects of anode and cathode tDCS can be inhibited by dextromethorphan (DMO), an N-methyl-D-aspartic acid (NMDA) receptor antagonist, which strongly suggests that NMDA receptors are involved in the two types of tDCS-induced neuroplasticity. On the contrary, the sodium (+) channel blocker carbamazepine (CBZ) selectively eliminated the anode effect. CBZ stabilizes the membrane potential based on the voltage, and the aftereffect of anode tDCS requires depolarization of the membrane potential. Consequently, it is believed that the polar drive changes in resting membrane potential are the key mechanism of the aftereffect of transcranial direct current induction, leading to changes in the spontaneous discharge rate and NMDA receptor activation (Liebetanz et al., 2002). The after effects of tDCS are also considered to involve non-synaptic mechanisms based on changes in neural membrane function. These changes not only reflect the local changes in ion concentrations, but also may be attributed to the changes in transmembrane proteins and relevant to [H(+)] electrolysis induced by a constant electric field (Ardolino et al., 2005).

### Trans-spinal direct current stimulation (tsDCS) enhance respiratory excitability

The mechanism underlying DiMEP enhancement after tsDCS may also involve changes in neurotransmission. During inspiration, glutamate drives the PMN pathway. NMDA and non-NMDA ionic glutamate receptors located in PMNs play an important role in the neurotransmission of inspiratory drive in adult rats (Chitravanshi and Sapru, 1996). However, activating NMDA rather than NMDA receptors is necessary for the formation and maintenance of ventilatory long-term facilitation (vLTF) in conscious rats (McGuire et al., 2008). A study found that both anode and cathode tsDCS can increase the *in vitro* release of glutamic acid analogue D-2,3-3H aspartic acid (Ahmed and Wieraszko, 2012).In contrast,expiratory neurons of the medulla oblongata-Rötzinger complex have a long-term descending inhibitory connection with PMNs (Merrill and Merrill and Fedorko, 1984). The γ-aminobutyric acid (GABA)-ergic system is closely related to respiratory motor control, and cathode tsDCS may act by directly inhibiting the spinal GABA-ergic system or by overexciting postsynaptic neurons (Ahmed, 2013). A study (Niérat et al., 2014) used paired-pulse technique to induce short interval intra-cortical inhibition (sICI) after tsDCS at C3-C5 levels, and found that sICI was not affected by tsDCS, suggesting that reducing GABA-mediated intra-cortical inhibition is not the cause for DiMEP enhancement after tsDCS. The residual effects induced by tsDCS do not occur at the brainstem or cortical level, but may mainly occur at the spinal cord level.

### Transcranial magnetic stimulation (TMS) enhance respiratory excitability

Some signaling pathways are believed to be related to respiratory plasticity. It is known that high-frequency rTMS (hfrTMS) strongly activates molecular pathways crucial for plasticity. A recent study demonstrated that acute hfrTMS can induce increased phosphorylation of the synaptic plasticity-related ribosomal protein S6 (rpS6). S6 phosphorylation is a downstream marker for the activation of multiple signaling pathways in neurons, including mitogen-activated protein kinase (MAPK)/extracellular signal-regulated kinase (ERK), ERK, kinase phosphatidylinositol 3-kinase (PI3K) and AKT/mammalian target of rapamycin (mTOR) signaling pathways, the combined action of which make synaptic and cellular changes the basis of plasticity (Gobert et al., 2008; Fujiki et al., 2020). Human and animal experiments have also confirmed that hfrTMS can induce increased expression of BDNF (Yukimasa et al., 2006; Gersner et al., 2011; Wang et al., 2011; Dall’Agnol et al., 2014). BDNF is believed to be involved in respiratory plasticity of PMNs (Baker-Herman et al., 2004). This factor also plays a crucial role in respiratory control during the injury after SCI (Lovett-Barr et al., 2012). In an animal experiment(Wang et al., 2011), 5-Hz rTMS was used daily for 5 days to improve rat BDNF–TrkB signaling by increasing the affinity of BDNF for tyrosine receptor kinase B (TrkB), resulting in an increase in TrkB phosphorylation. The increase in BDNF–TrkB signaling was accompanied by an increased correlation between activated TrkB and NMDA receptors (NMAR). In normal human subjects, 5-d rTMS of the motor cortex reduced the resting motor threshold, which is related to the enhancement of the BDNF–TrkB signaling pathway in lymphocytes and TrkB-NMDAR correlation. These results indicate that rTMS promotes the function of BDNF–TrkB-NMDARs in the cortex and lymphocytes. TMS can also alter respiratory excitability by reducing the intensity of inhibitory synapses. Studies (Michel-Flutot et al., 2021) have found that a single 10-Hz rTMS in anesthetized rats can induce an increase in the excitability of the phrenic neural network. Intravenous injection of GABA_A_ and GABA_B_ receptor agonists before treatment with 10-Hz rTMS can eliminate the enhanced PMN excitability, indicating that a single high-frequency rTMS protocol at 10 Hz can alleviate the increased PMN excitability through local GABA-ergic-mediated inhibition. In *in vitro* experiments, it was also observed that 10-Hz magnetic stimulation could induce a decrease in the postsynaptic GABA-ergic synaptic strength of neurons (2–4 h after stimulation), which was Ca (2+)-dependent and accompanied by remodeling of the postsynaptic gephyrin scaffold (Lenz et al., 2016). Additionally, TMS may induce respiratory plasticity by reducing inflammatory responses. A recent study found that 10-Hz rTMS treatment could reduce inflammation of the spinal cord (C1-C3) in rats with C2 hemisection (reduced CD68 and Iba1 labeling) and accelerate the intracellular plasticity of PMNs, thereby enhancing the respiratory descending fibers in the ventrolateral funiculus (increased GAP-43-positive fibers), indicating that chronic high-frequency rTMS can improve respiratory dysfunction after cervical spinal cord injury and induce neuronal plasticity by reducing harmful post-traumatic inflammatory processes (Michel-Flutot et al., 2022).

### Safety and tolerability of TMS/tDCS

The most commonly reported adverse events of TMS are transient or mild headaches and local discomfort at the site of irritation (Hao et al., 2013). The only potentially serious side effect is seizures. It is now certain that the risk is very low (Rossi et al., 2009, 2021). The most commonly reported effects of tDCS are tingling and itching under electrodes, headache, and fatigue (Poreisz et al., 2007; Fertonani et al., 2015). Unlike rTMS, no cases of induced seizures have been reported to date. Skin damage has occasionally been reported (Palm et al., 2008), in most cases,it is associated with program defects, such as dryness of the contact medium under the electrode. In summary, the commonly used tDCS/TMS protocol is safe and well tolerated.

### Challenges and open issues faced

The application of rTMS/tdc in respiratory function is still investigational. The study of the effect of TMS on respiratory muscle function is challenging. The effectiveness of TMS-MEP mainly depends on the appropriate positioning of electromyographic electrodes and the control of background muscle activity and noise. Through this technique, the signals recorded during non-specific muscle contractions (such as contraction generated by cortical magnetic stimulation) are the sum of the electrical activities generated by all muscles below the electrodes. Therefore, we may question whether these signals indeed originate from the diaphragm and there are several factors that support diaphragmatic origin, including the latency from the cortex to the diaphragm, changes in intrathoracic and abdominal pressure or abdominal circumference and electrode positioning (Similowski et al., 1996). Compared to the diaphragm, the cortical center of the expiratory muscle (abdominal muscle) is more difficult to locate, resulting in a smaller MEPs amplitude. Other challenges include ensuring that the TMS coil is correctly positioned and maintained in the cortical area of interest throughout the entire TMS process. However, TMS neuronavigation devices have been proven to effectively reduce this potential confoundingly (Caulfield et al., 2022; Nieminen et al., 2022). Moreover, respiration-related indicators of TMS still have significant individual differences and lack standard values forhealthy individuals. Clinical trials with a large sample size are needed to establish standard values. When applying tDCS in the clinical population, it should be considered that tDCS has a brain-state-dependent effect as a neuroregulatory intervention (Thirugnanasambandam et al., 2011; Antal et al., 2014). Concurrent drug therapy can further alter the effect of tDCS, which is an important consideration in all tDCS studies (Stagg and Nitsche, 2011). Another problem with tDCS is that the spatial resolution is too low to accurately stimulate functional subdivisions of the cortical regions. Improving tDCS focus is an important direction in the future (Woods et al., 2016). For rTMS/tDCS mode, in addition to identifying ideal cortical regions to maximize the therapeutic response (which may require a combination of clinical, neuroimaging and neurophysiological information), it is important to optimize the stimulation mode, so as to best regulate the activity of these regions and move them toward the expected direction. Future work and exploration will focus on the combination of neuroregulation with other therapeutic interventions (including respiration training and exercise therapy), simultaneous central and peripheral interventions, as well as the application of closed-loop theory. The closed-loop theory, which was first proposed in 2016 and refers to combining central intervention measures with peripheral intervention measures to form a positive feedback loop to promote motor function rehabilitation in stroke patients (Jia, 2016, 2022).

## Conclusion and future directions

Although TMS/tDCS is not currently approved as adjuvant treatments for respiratory dysfunction, considering the clinical importance of respiratory dysfunction and the lack of treatment means, it is of great significance to further explore the therapeutic potential of this neuromodulation strategy in respiratory dysfunction. We suggest four fields for future research on TMS in central respiratory dysfunction. These studies will ultimately help to develop better neuromodulation-based interventions for patients: (1) Optimizing animal models. At present, most preclinical studies mainly involve rodents, whose respiratory physiology is very different from that of humans, but in the future, preclinical models can be considered in mammals that are more similar to human respiratory physiology, such as cats, rabbits and so forth. (2) TMS-MEP was used to determine respiratory corticospinal tract conduction and cortical excitability abnormalities and to further clarify the disorders of respiratory neural circuits in different physiological and pathological states in combination with techniques such as EEG, providing more accurate neural targets for non-invasive regulation of respiratory dysfunction. (3) rTMS/tDCS was used to regulate respiratory neural circuits to determine the impact of this neuromodulation on respiration-related biological and clinical parameters. Before and after acute rTMS/tDCS, neuroimaging and/or neurophysiological evaluation, as well as clinical evaluation can elucidate the causal role of respiratory neural circuits in specific symptoms or behavioral development to better guide subsequent therapeutic intervention based on neuromodulation. Functional neuroimaging, especially functional imaging including functional connectivity measurement, has been used to prospectively determine non-invasive neuroregulatory targets for future therapeutic interventions. (4) Combining neuroimaging, neurophysiology and clinical measures relevant to respiration-targeted neural circuits to better predict and track outcomes in clinical trial studies.

## Author contributions

LL and XiaC conceived and organized the manuscript. LL, XiaC, and JY researched literature. JN and XinC proofread the manuscript. All authors contributed to the manuscript and approved the submitted version.

## Funding

This work was supported by the Fujian Provincial Health Technology Project (grant number: 2020TG016), the Startup Fund for Scientific Research of Fujian Medical University (grant number: 2020QH1017), and the Fujian Provincial Health Youth Research Project (grant number: 2021QNA024).

## Conflict of interest

The authors declare that the research was conducted in the absence of any commercial or financial relationships that could be construed as potential conflicts of interest.

## Publisher’s note

All claims expressed in this article are solely those of the authors and do not necessarily represent those of their affiliated organizations, or those of the publisher, the editors and the reviewers. Any product that may be evaluated in this article, or claim that may be made by its manufacturer, is not guaranteed or endorsed by the publisher.
