# Supplementary material for: Novel role for non-invasive neuromodulation techniques in central respiratory dysfunction
Source: Front Neurosci. 2023 Aug 23;17:1226660. doi: 10.3389/fnins.2023.1226660 (PMC10480838; doi:10.3389/fnins.2023.1226660)
Supplement: Supplementary file 1 [file Table_1.DOCX]

Supplementary Table 1 Studies of TMS as a tool for assessing respiratory plasticity

| Reference | Subject characteristics | Coil  Site  Muscle | index | Main finding | Suggestion | |
| --- | --- | --- | --- | --- | --- | --- |
| Harraf 2008 | Patients with acute stroke (n = 15)  Controls (n = 16) | Double-cone  Vertex/bilateral hemispheric abdominal muscular cortex  RA/EO/DI | MEP  PE max  PCFR  Cough, and changes in Pdi and Pgas under TMS, TwT 10 and BAMPS | No MEPs were elicited by TMS in the abdominal muscles of the injured hemisphere  Stroke patients showed a reduction in PCFR, cough Pgas, TMS Pgas at the vertex.Pgas was lower in the injured hemisphere than the uninjured side under TMS. | Ischemic cortical injury is correlated with expiratory muscle weakness and may cause cough in stroke patients | |
| Duguet 2006 | Long-term ventilator dependence (n = 11)  Ventilator use less than 12 w (n = 16) | Circular coil  Vertex  DI/APB | Cx-MEP  Sp-MEP | Six completely ventilator-dependent patients had no diaphragmatic responses. Another 10 patients restored autonomous ventilation completely (n = 9) or partially (n = 1). | | The electrophysiological study of the diaphragm under TMS is helpful to predict the recovery of central respiratory paralysis within 1 year |
| Miscio 2006 | ALS  (n = 14) | Circular coil  Vertex  DI | FVC, FEV1, PEF, blood gas analysis  MIP/MEP, Pdimx  Cx-MEP/Sp-MEP  CMCT | Seven patients had a decrease in Pdimx, and 8 patients showed a decrease in MEPs. Four patients presented delayed Sp-MEPs. Cx-MEPs were not elicited1 in patients. The correlations of Cx-MEPs and CMCT with any respiratory measurement were not significant. | Cortico-diaphragmatic research is a sensitive method to reveal subclinical diaphragmatic injury, although not correlated with respiratory measurements. | |
| Miscio 2003 | MS  (n = 26) | Circular coil  Vertex  DI/Abd | EDSS, FDS.  FVC, FEV1, PEF  Cx-MEP  CMCT  PN-CMAP | Cx-MEP latency and CMCT were prolonged in the Dia of 31% and 23% patients, as well as in the Abd of 76% and 79% patients. PN-CMAPs were normal. There was no significant correlation between TMS results and respiratory measurements. | The cortico-diaphragmatic pathways are only damaged in a few MS patients. | |
| Urban PP 2002 | Healthy volunteers (n = 30)  Cerebral infarction with injured pyramidal tract (n = 31) | 8-shaped coil  2-3 cm lateral and 2 cm anterior to the vertex  DI | MEPs  CCT | All healthy subjects experienced contralateral responses during spontaneous inspiration, and only 18 (right) and 21 (left) subjects presented ipsilateral responses during spontaneous inspiration. Most of the 31 patients with cerebral infarction patients had abnormal DiMEP | | The voluntary activation of respiratory muscles is mainly mediated by the contralateral motor cortex; Descending cortical-respiratory projections are located within the pyramidal tract; 3. Cortical-respiratory projections of the respiratory muscles are often involved in patients with acute stroke-induced hemiplegia. |
| Khedr 2000 | Patients with stroke (n = 34)  Controls (n = 25) | 8-shaped coil  3 cm lateral to the midline, and 2-3 cm in front of the auricular plane  DI | MEPs  CCT  CMAPs | Twenty-four (70.5%) patients had abnormal MEP latency, CCT, CMAP amplitude and excitability threshold in the affected hemisphere. The levels of hypoxia, hypocapnia and blood bicarbonate saline in patients with hemiplegia were significantly higher than those in the control group. | Patients with acute stroke may experience central diaphragmatic dysfunction, which is closely related to the occurrence of hypoxia. | |

EDSS = Expanded disability status scale; FDS = fatigue descriptive scale; FEV_1_ = forced expiratory volume at the first second; FVC = Forced vital capacity; MIP/MEP = maximal inspiratory and expiratory pressure at mouth;PCFR = peak voluntary cough flow rates; Pdi = transdiaphragmatic pressure;PEmax = maximum static expiratory pressure; PEF = peak expiratory flow ; Pgas = intragastric pressure; SNIP = sniff effort from FRC level;
