# Supplementary material for: Novel role for non-invasive neuromodulation techniques in central respiratory dysfunction
Source: Front Neurosci. 2023 Aug 23;17:1226660. doi: 10.3389/fnins.2023.1226660 (PMC10480838; doi:10.3389/fnins.2023.1226660)
Supplement: Supplementary file 2 [file Table_2.DOCX]

Supplementary table 2 Preclinical studies on assessment and neuromodulation of respiration

| Reference | Animal model and grouping | Device and stimulation site | Protocol | Main finding | |
| --- | --- | --- | --- | --- | --- |
| Michel-Flutot 2022 | Male SD rats with C2 hemisection  6 groups:  10 Hz (7 d, 1 month, 2 months)  Sham stimulation (7 d, 1 month, 2 months) | TMS (MAGPRO R30)  8-shaped coil  (Cool-B65)  6 cm from the caudal end of the anterior fontanelle | 10 Hz, 9 columns of 100 bidirectional pulses, 50% maximum output, 900 times, interval 30 s | Chronic high-frequency can improve respiratory dysfunction after cervical spinal cord injury, and reduce harmful traumatic inflammatory processes to induce neuronal plasticity | |
| Michel-Flutot 2021 | Male SD rats, divided into 8 groups  Sham stimulation group, 3 Hz group, 10 Hz group, 30 Hz group, Sham rTMS + clonazepam group, Sham rTMS + baclofen group, 10 Hz rTMS + clonazepam group, 10 Hz rTMS + baclofen group | TMS (MAGPRO R30)  8-shaped coil  (Cool-B65)  6 cm from the caudal end of the anterior fontanelle | 9 columns of 100 bidirectional pulses, 900 times, interval 30 s | 10-Hz TMS induces a sustained increase in the excitability of PMNs, and intravenous injection of GABA_A_ and GABA_A_ receptor agonists before 10-Hz rTMS treatment can eliminate enhanced PMN excitability | |
| Vint 2016 | Male SD rats with C2 hemisection  Divided into 3 groups: 1 h after injury group  7 d after injury group  Sham surgery group | TMS (MAGPRO X100)  8-shaped coil  (CB60)  6 cm from the caudal end of the anterior fontanelle | Monopulse TMS | Diaphragmatic reorganization is induced, DiMEP decreases on the non-injured side rather than the injured side, and ipsilateral PMN excitability increases |  |
| Vint 2014 | Male SD rats, 2 groups  Magnetic shield group  Non-shield group | TMS (MAGPRO X100)  8-shaped coil  (CB60)  6 cm from the caudal end of the anterior fontanelle | Monopulse TMS | TMS-induced DiMEPs can be successfully recorded in rats and may be used to evaluate supraspinal respiratory plasticity |  |
| Lee 2021a | SD rats with left mid-cervical spinal cord injury  6 groups  Non-injured group  Spinal cord contusion group  (acute injury group, subchronic injury group and chronic injury group) | CMS (MAGPRO R30)  8-shaped coil (Cool-B65)  20 cm from the caudal end of the anterior fontanelle | Monopulse CMS  The anterior fontanelle is located 30 mm left to the coil center, midline, and 30 mm left | When the rat head is placed 30 mm right or left to the coil center, a single magnetic stimulation can induce significant DiMEPs in non-injured animals. In the acute stage, cervical magnetic stimulation reduces the threshold of DiMEP and enhanced its amplitude. |  |
| Lee 2021b | SD rats with left mid-cervical spinal cord injury6 groups  Non-injured group (at the same time point)  Spinal cord contusion group  (acute injury group, subchronic injury group and chronic injury group) | CMS (MAGPRO R30)  8-shaped coil (Cool-B65) | Monopulse, the coil is located left  The distance between the anterior fontanelle and the coil center is 20, 30 and 40 mm, respectively | the DiMEPs of the left diaphragm is higher and its occurrence is earlier than those of the right. Caudal cervical magnetic stimulation generates higher DiMEPs compared with rostral cervical magnetic stimulation. |  |
