# Supplementary material for: Novel role for non-invasive neuromodulation techniques in central respiratory dysfunction
Source: Front Neurosci. 2023 Aug 23;17:1226660. doi: 10.3389/fnins.2023.1226660 (PMC10480838; doi:10.3389/fnins.2023.1226660)
Supplement: Supplementary file 3 [file Table_3.DOCX]

Supplementary table 3. Clinical studies on neural modulation of respiration

| Reference | Population | Stimulation device | Site | Protocol | Observation indexes | Main finding |
| --- | --- | --- | --- | --- | --- | --- |
| Cao HY et al.  2022 | Stroke patients with respiratory dysfunction  N = 60 | TMS (MagPro R30)  8-shaped coil (B65) | 3 cm lateral to the midline, and 2-3 cm in front of the auricular plane | 5Hz (4200)  10 min | DT, DFT. DM  FVC, FEV1, FEV1/FVC, PEF, MVV  ADL | TMS can improve pulmonary function after acute ischemic stroke |
| Carvalho  2021 | SCI patients  N = 2 | tDCS | In one patient  Anode: SMA  Cathode: Supraorbital region  PES: four times at the abdomen, and four times at the chest and abdomen  In another patient  PES: four times at the abdomen | 20 min,  2 mA,  15 times | MIP  MEP  PCF  DM, DFT | Extubation is successful, and coughing ability is improved |
| Nierat  2015 | Healthy individuals  N = 7 | TMS  8-shaped coil  Neuro-navigation | 1 cm to the forefront of the site where right AH and SMA can be induced | CTBS (200 ms, 3times, 50 Hz, 600 Hz)  5 Hz(interval 50 s)  Sham stimulation | Ti, TE, TT,  TI/TT,  VT, F | After sham stimulation, there is no significant change in respiratory pattern during inspiratory load. The 5 Hz pre-treatment can shorten inspiratory time, reduce tidal volume, and decrease ventilation. Inhibitory stimulation does not affect ventilation, but prolongs expiratory time. |
| Nierat  2014 | Healthy individuals  N = 22 | tDCS | One electrode is placed at the spinal cord segments C3-C5, and another at the midline of the anterior edge of the neck  Interchange of positions | Anode tDCS  Cathode tDCS  2.5 mA  15 min | DiMEP  respiratory pattern evaluation | Anode and cathode induce a progressive increase in MEP amplitude at least 15 after current termination, and cathode induces a sustained increase in tidal volume rather than anode. |
| Azabou  2013 | Healthy individuals  N = 12 | tDCS | Anode tDCS: Anode at the primary motor cortex of the left diaphragm  Cathode tDCS: Above the right orbit  Cathode: Opposite position  Sham stimulation | 2 mA  10 min | Right diaphragm | Direct current stimulation reduces the excitability of the diaphragmatic corticospinal pathway regardless of polarity. No changes are found under sham stimulation. |
| Laviolette  2013 | Healthy individuals  N = 12 | TMS  8-shaped coil (70 mm)  Double-cone coil that cannot elicit DiMEPs (90 mm)  Neuro-navigation | 1 cm to the forefront of the site where AH can be induced | cTBS  3 times per 200 ms, 50 Hz pulse (600 pulses)  5 Hz  500 pulses, interval 50 s | FDI-MEP  AH-MEP  DiMEP | SMA can bi-directionally regulate the corticospinal pathway of the diaphragm. |
| Raux  2010 | Healthy individuals  N = 11 | TMS  8-shaped coil (70 mm)  Double-cone coil that cannot elicit DiMEPs (90 mm)  Neuro-navigation | SMA | 5 Hz, 10 s, 10 times, interval 50 s  10 Hz, 5 s, 10 times, interval 55 s  1 Hz, 20 min (1200) | DiMEP | It can increase diaphragmatic corticospinal excitability, and is functionally correlated |

ADL = activities of daily living; AH = abductor hallucis ;DT = diaphragmatic thickness; DTF = diaphragmatic thickening fraction; DM = diaphragmatic mobility; f = breathing frequency ;MVV = maximum ventilatory volume; VT = Tidal volume; TI = Inspiratory time; TE = expiratory time; TT = total time;TI/TT = duty cycle;
